# Separation of powers: A key feature underlying the neuroprotective role of Retromer in age-related neurodegenerative disease?

**DOI:** 10.1016/j.ceb.2025.102516

**Published:** 2025-04-19

**Authors:** Brett M. Collins, Peter J. Cullen

**Affiliations:** 1 The University of Queensland, Institute for Molecular Bioscience, St Lucia, Queensland, 4072, Australia; 2 School of Biochemistry, Biomedical Sciences Building, Faculty of Health Sciences, University of Bristol, Bristol BS8 1TD, UK

## Abstract

The retromer complex was discovered in *Saccharomyces cerevisiae* as a multiprotein, pentameric assembly essential for recycling of integral membrane cargo proteins through the endosomal network [1,2]. We now understand how retromer is assembled, its membrane architecture, and how it selects proteins for recycling [3–6]. Conserved across eukaryotes, analyses have revealed retromer’s role in organism development, and homeostasis and has linked retromer defects with age-related Alzheimer’s disease and Parkinson’s disease and other neurological disorders [3,5,7]. Indeed, stabilizing retromer function is now actively considered a therapeutic strategy [8]. Here, we reflect on its structural and functional evolution rather than overviewing retromer biology (see, *e.g*. [5,7]). Specifically, we clarify the organization of the human retromer to provide greater focus for future research, especially within the context of retromer’s function in neuroprotection.

## Introduction

In humans, thousands of integral membrane cargo proteins and associated proteins and lipids undergo flux through the endosomal network. On entering the network, each cargo faces a fate decision: either sorting and transport to the lysosome for degradation and loss to the cell or retrieval and recycling for reuse at organelles that include the cell surface, the autophagic and biosynthetic pathways, and, in specialized cells, lysosome-related organelles [1–3]. Each fate decision is organized around two distinct nanodomains on the endosomal limiting membrane: the degradative subdomain and the retrieval subdomain [4]. The degradative subdomain is principally defined by enrichment of ESCRT proteins and the inward budding of vesicular profiles into the endosomal lumen. In contrast, the retrieval subdomain is defined by (i) cargo-enriched tubularevesicular profiles and clusters that emanate from the limiting membrane into the cytoplasm (These form the carriers and/or conduits that transport retrieved cargo for recycling.); (ii) a highly localized network of branched, filamentous actin that is highly dynamic, with too little and too much actin both disrupting subdomain organization and cargo retrieval and recycling [5,6]; and (iii) several multiprotein complexes that include Retriever and the CCC complex (which associate to form the Commander super-assembly) [7–11], the actin-polymerizing WASH complex [12,13], membrane-tubulating sorting nexin (SNX)–Bin/Amphiphysin/Rvs (BAR) proteins [14], and, of course, retromer. An important distinction to note is that the WASH, Retriever, and CCC complexes are not expressed in yeast [8,15], immediately suggesting a fundamental difference between the organization and regulation of the human (and metazoan) retrieval subdomain with any corresponding subdomain in yeast.

### The yeast retromer–SNX–BAR pentamer—the exception, not the rule

Retromer is a heterotrimer of Vps26, Vps35, and Vps29, which for the yeast retromer forms a stable pentameric assembly with a heterodimer of Vps5 and Vps17, members of the SNX family with BAR domains [16,17,14]. While the self-assembling membrane-associated Vps5:Vps17 heterodimer can promote biogenesis of tubular–vesicular transport carriers, it recruits the Vps26:Vps35:Vps29 heterotrimer via a high-affinity interaction which stabilizes the membrane-assembled pentameric complex [18,19]. The Vps26:Vps35:Vps29 heterotrimer additionally acts to select integral proteins for entry into the retrieval and recycling pathway [16]. By coordinating these processes, cargo recognition with transport carrier biogenesis, the yeast retromer–SNX–BAR pentameric complex displays the hallmarks of a coat complex [16,17].

**Figure 1:**
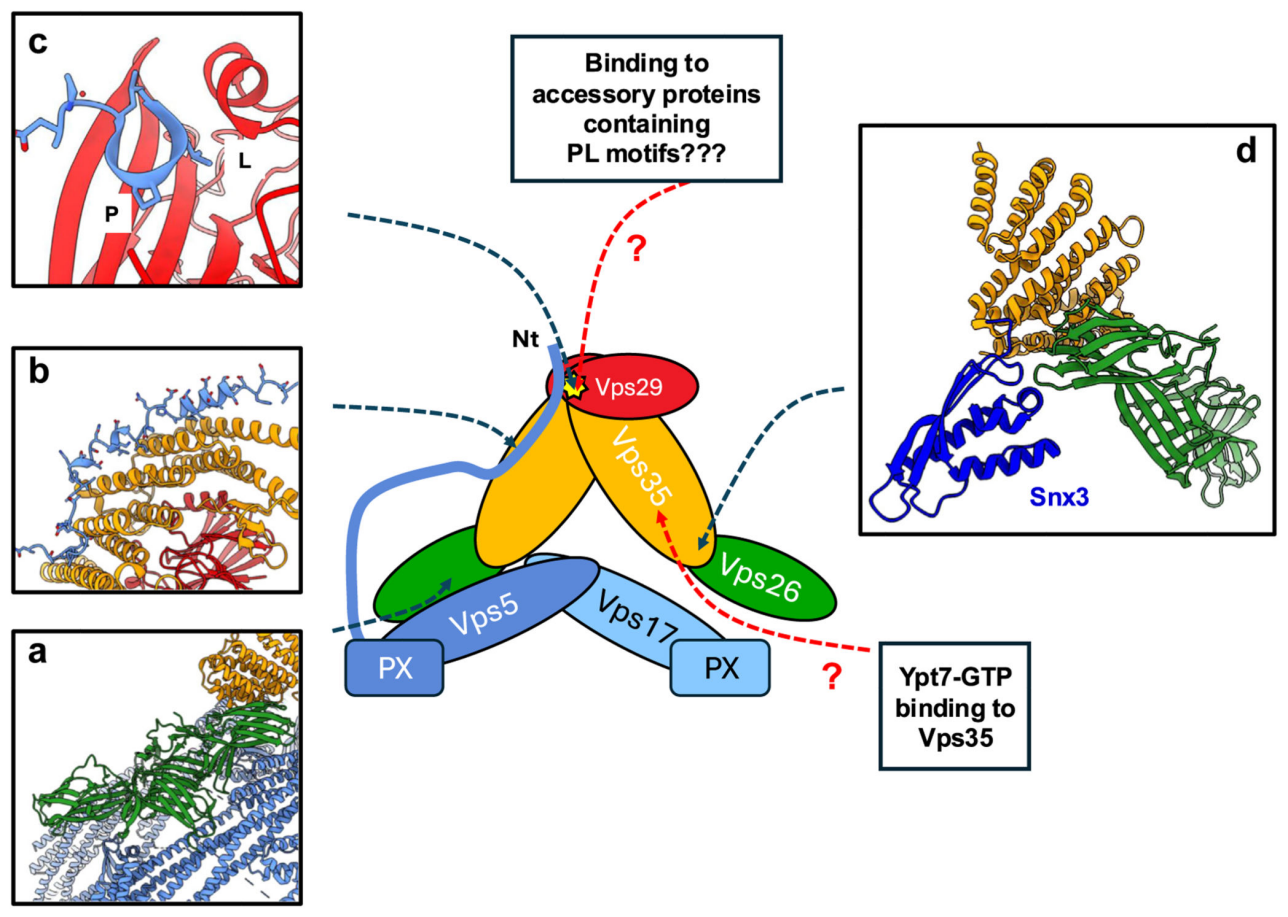
The yeast retromer–SNX–BAR pentameric assembly. As discussed, the structural features that drive assembly of the stable pentameric retromer–SNX–BAR assembly have been resolved. This raises several important questions. First, does the binding of Vps5 to Vps26 **(a)**, and the Vps5 amino-terminal (Nt) extension for Vps35 and the PL motif-binding pocket in Vps29 **(b**,**c)** preclude other proteins from utilizing this site as observed in higher organisms? In a single pentameric complex of Vps35–Vps26–Vps29–Vps5–Vp17 the Vps5 amino-terminus would occupy the sole PL motif–binding site; however, with a dimeric arch of retromer trimers, the PL motif–binding site in one Vps29 would be a free to either be occupied by the PL motif from an adjacent Vps5 protein in a membrane-assembled lattice or to engage as yet to be identified accessory proteins similar to the metazoan Retromer complex. Secondly, as cryo-ET studies have established that retromer associates with membrane-bound Snx3 **(d)** and Vps5-Vps17 using overlapping sites in Vps26 **(a)** (*i.e*. in principle, the assembly of these lattices would be mutually exclusive), how is the high-affinity interaction observed in the retromer–SNX–BAR reconciled with retromer’s ability to also interact with the Snx3 cargo adaptor? There are several possible and not mutually exclusive answers. First, the concentration of the retromer trimer may be sufficiently high in a yeast cell or within a specific membrane domain to interact with both Snx3 and Vps5–Vps17 simultaneously. Secondly, it is possible that the interaction of Vps5–Vps17 with retromer is regulated either by other proteins or posttranslational modification, which could allow the retromer to dissociate and form an alternate complex with Snx3. Lastly, as with the PL motif occupancy of Vps29 (see previous text), in a single dimeric arch of the Vps35–Vps26–Vps29–Vps5–Vp17 pentameric complex, there would be a free Vps26 subunit to engage with an Snx3 cargo adaptor, although whether Snx3 and the Vps5–Vps17 proteins can exist in overlapping coat structures remains untested. BAR, Bin/Amphiphysin/Rvs; PL, Pro-Leu; SNX, sorting nexin.

Building on this work, we now understand the mechanism for Vps5:Vps17 self-assembly and biogenesis of tubular transport carriers, these proteins possess membrane-remodeling BAR domains [14,20–22]), and we are beginning to obtain an understanding of the mechanisms of sequence-dependent integral protein recognition by the Vps26:Vps35:Vps29 heterotrimer [23]. A key breakthrough has been the elucidation of the architecture of membrane associated retromer [24]. This entails assembly of dimeric arches of Vps26:Vps35:Vps29 organized around a membrane-proximal Vps26:Vps35 base with membrane-distal Vps35:Vps29 regions constituting the dimeric interface at the top of individual arches. Neighboring dimeric retromer arches associate to form a loosely organized pseudohelical array bound to the membrane-associated Vps5:Vps17 heterodimer [24] (Figure 1).

Humans express a stable VPS26:VPS35:VPS29 heterotrimer [25], which forms an analogous arch-like architecture on membranes [26]. Added complexity arises from two VPS26A and VPS26B orthologous genes [25,27] and at least three VPS29 orthologs—generally speaking, the functional significance of these orthologs remains poorly understood [25,28]. Similarly, humans express SNX1 and SNX2 as orthologs of Vps5, and SNX5 and SNX6 as Vps17 orthologs (SNX6B, also known as SNX32, is also expressed in human neurons, but for simplicity, we consider these under one term ‘SNX6.’) [25,29]. These orthologs retain the yeast Vps5:Vps17 heterodimeric rule; either one of SNX1 or SNX2 assemble with either one of SNX5 or SNX6 [29,30].

Yeast and metazoans do share an additional retromer assembly composed of the Vps26:Vps35:Vps29 heterotrimer associated with the non−eBAR domain SNX, Snx3p in yeast and SNX3 in humans [31–33]. Endosome-associated Snx3p/SNX3 recruits Vps26:Vps35:Vps29 with sequence-dependent cargo recognition being achieved through the interface of Vps26 with SNX3 [31–34]; Vps26:Vps35:Vps29 can also be recruited to endosomes through binding to active Ypt7 in yeast and RAB7 in humans [35,36]. Cargo recognition facilitates endosomal association and formation of an SNX3–retromer cargo retrieval and recycling complex which, through assembly of retromer arches, forms a loosely organized membrane bound helical array that, at least *in vitro*, drives tubule biogenesis and the sorting of cargo that includes the iron transporter DMT1-II [26,37–40].

### Evolution of the retromer blueprint

The yeast retromer–SNX–BAR pentameric complex provided the dogma for the initial analysis of the equivalent proteins in humans and metazoans. The yeast retromer–SNX–BAR complex is assembled through two core interactions; Vps26 directly interacts with Vps5 [24,41], and the intrinsically disordered amino-terminal region of Vps5 presents a bipartite sequence that directly binds to Vps29 and Vps35 [42,19] (Figure 1). A key element of this bipartite sequence is a Pro-Leu (PL) motif from Vps5 that binds into a hydrophobic groove in Vps29—of which more later [42,19]. In addition, Vps35 engages with Leu-Phe−containing sequences in Vps5 that enhance the affinity of the interaction [42,19].

In humans, while there is some evidence of a direct interaction between VPS26:VPS35:VPS29 and the SNX1/SNX2:SNX5/SNX6 heterodimer [25], most studies point to this potential interaction as being, at best, of low affinity and highly transient. For instance, while cryo-electron tomography (cryo-ET) and subtomogram averaging of membrane tubules coated with Vps5 and Vps26:Vps35:Vps29 has revealed stable retromer arches [24], the human VPS26:VPS35:VPS29 heterotrimer is unable to form a complex with the membrane-associated SNX1:SNX5 coat [43,44].

**Figure 2:**
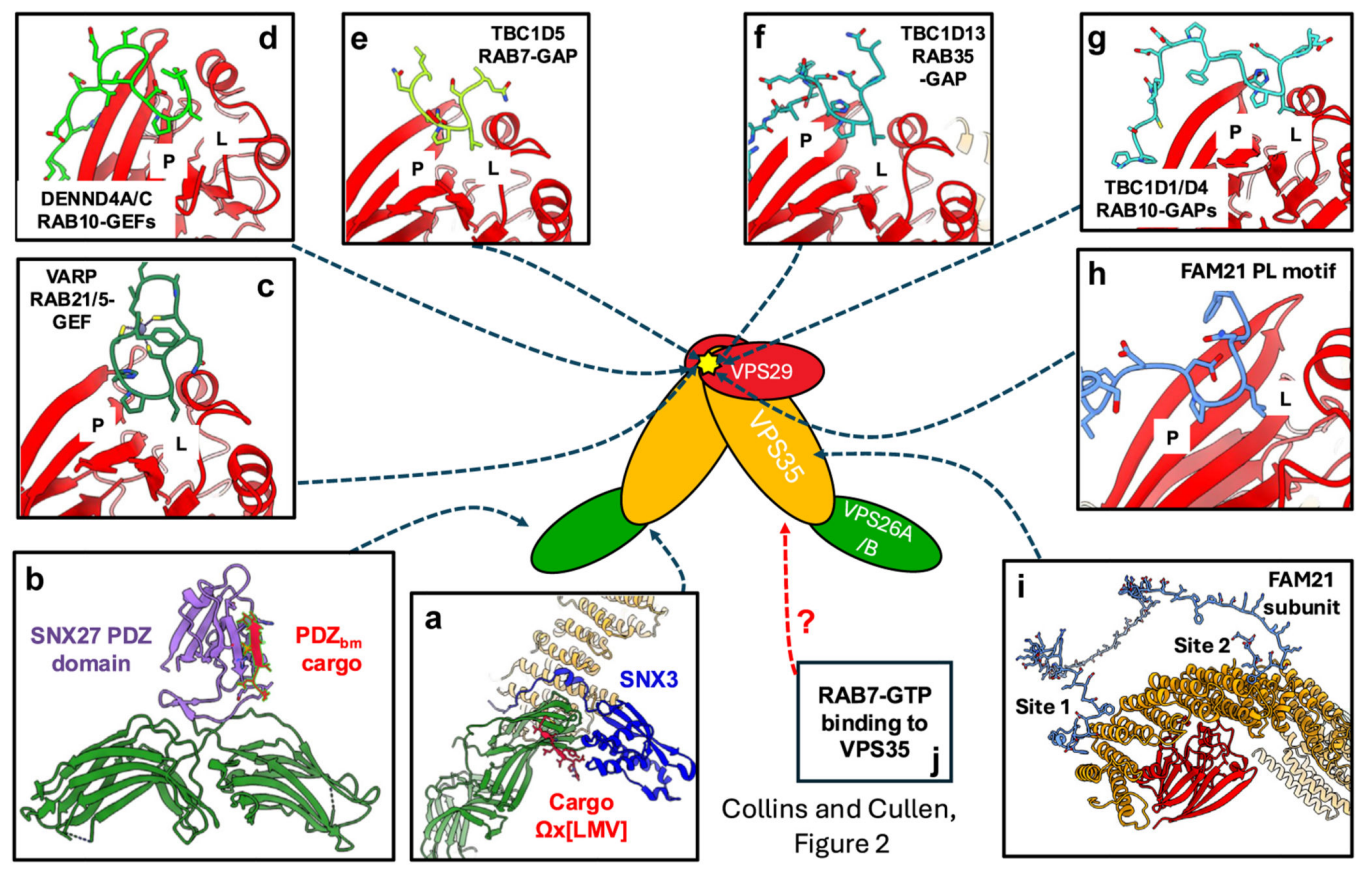
The human and metazoan Retromer assembly. Retromer associates with the cargo adaptors SNX3 and SNX27 to provide routes for sequence-dependent cargo recognition and sorting **(a**,**b)**. Separation of retromer from the ESCPE-1 complex has expanded the repertoire of PL motif–containing accessory proteins associating with the Leu hydrophobic pocket (yellow star) in VPS29 **(c–g)**, including a variety of RAB regulators controlling specific endosomal RAB GTPases. Additionally, the FAM21 subunit of the WASH complex presents a PL motif for binding to VPS29 and associates with two sites, site 1 and site 2, on VPS35 to associate the entire WASH complex with the retromer retrieval subdomain **(h**,**i)**. In addition to SNX3 recruiting retromer to endosomes **(a)**, active RAB7 also enables retromer endosomal recruitment, although the structural basis of this mechanism remains unresolved **(j)**. PL, Pro-Leu; SNX, sorting nexin.

**Figure 3:**
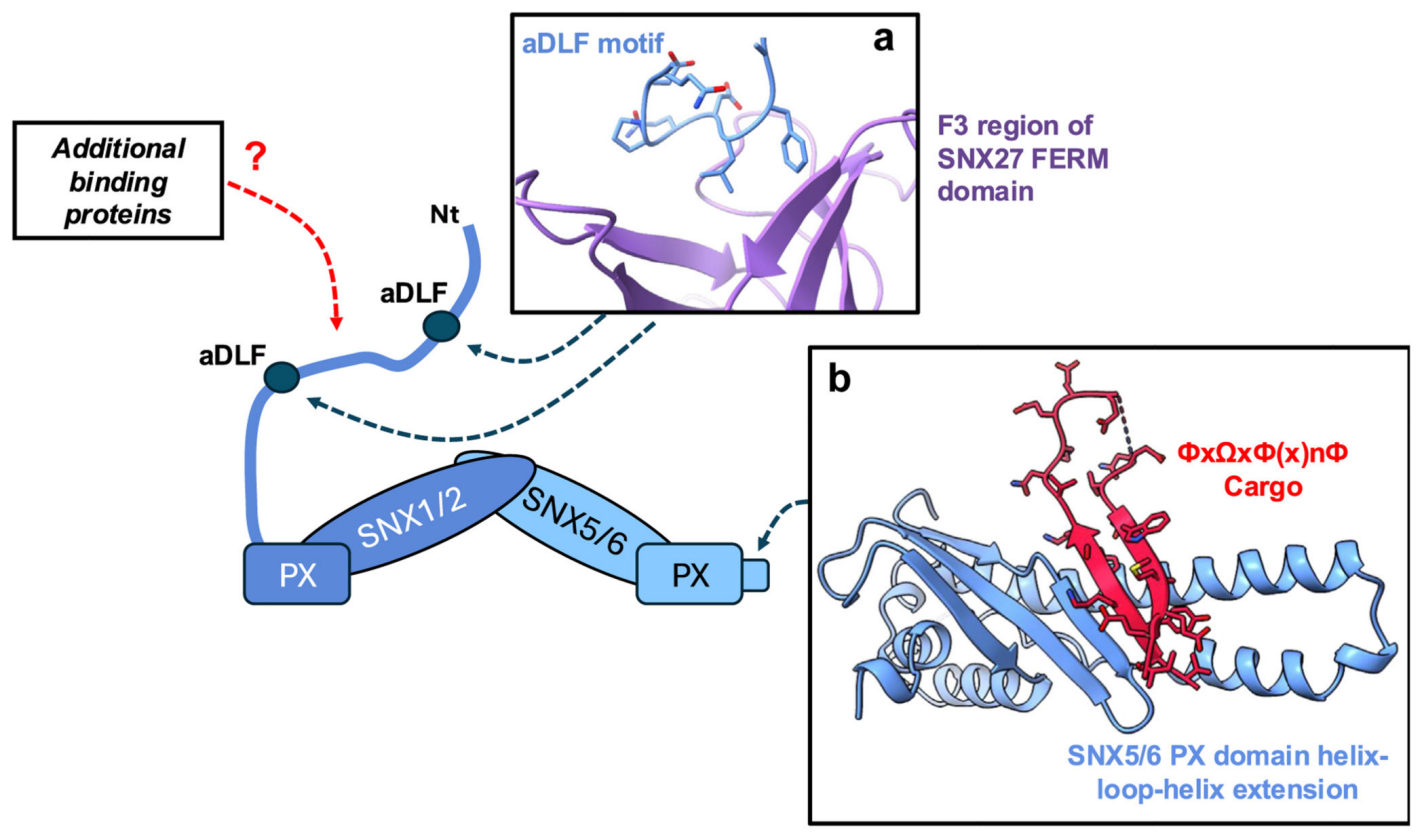
The ESCPE-1 complex. The amino-terminal unstructured region of SNX1/2 directly associates with the SNX27–retromer assembly **(a)**—it lacks the PL motif present in the corresponding region of yeast Vps5 that aids assembly of the retromer–SNX–BAR pentameric assembly (Figure 1c). Through the evolution of a helix-loop-helix extension, the PX domain of SNX5/6 has acquired the ability to directly bind selective cargo proteins **(b)**, a feature absent in the yeast Vps17 equivalent. PL, Pro-Leu; SNX, sorting nexin.

Consistent with this, the intrinsically disordered aminoterminal regions of SNX1 and SNX2 lack elements of the conserved bipartite motif that in Vps5 stabilizes the pentameric retromer–SNX–BAR assembly through binding to Vps29 and Vps35 [42,19]. Functional analysis of endosomal sorting in human cells is also consistent with SNX1/SNX2:SNX5/SNX6 heterodimers acting independently of the VPS26 A/B:VPS35:VPS29 heterotrimer [45,46]. These data support that in humans, the VPS26 A/B:VPS35:VPS29 heterotrimer and the SNX1/SNX2:SNX5/SNX6 heterodimer have evolved into two distinct complexes that do not form a stable pentameric association (We suspect this is common to Retromer across metazoans.) (Figures 2 and 3). To signify this fundamental difference, a nomenclature has evolved, where in humans (and metazoans), the term ‘Retromer’ refers to the VPS26 A/B:VPS35:VPS29 heterotrimer [46], while the SNX1/SNX2:SNX5/SNX6 heterodimer has been termed the ESCPE-1 complex [47,48]. How has this ‘separation of powers’ come about, and why is such a separation so important in understanding human Retromer biology?

### VPS29−a keystone subunit

VPS29 is the smallest subunit of retromer and is the only subunit shared with the related Retriever complex [8]. VPS29 contains two highly conserved hydrophobic surfaces [49,50]. One surface is buried in the interfacial interaction with the carboxy-terminal portion of the VPS35 α-solenoid; the other, on the opposite side, contains a solvent exposed hydrophobic groove. In the yeast pentameric retromer, it is this solvent-exposed groove that binds to the PL motif within Vps5 to aid pentameric complex assembly [42,19]. The lack of an equivalent PL motif in the human SNX1 and SNX2 orthologs of Vps5 provides part of the molecular explanation for the lack of formation of a stable human pentameric retromer assembly (as discussed earlier). Moreover, this also liberates the solvent-exposed PL-binding site in VPS29 for association with an array of functionally diverse proteins that include the RAB7 GTPase-activating protein (GAP) TBC1D5 [51,52], the RAB21 guanine-nucleotide exchange factor (GEF) VARP [53], the FAM21 subunit of the actin-polymerizing WASH complex [54,55], the Retriever VPS35L subunit [9–11], and the RAB10 GEFs DENND4A and DENND4C, the RAB10 GAPs TBC1D1 and TBC1D4, and the RAB32 GAP TBC1D13 [56]. All these accessory proteins present PL motifs for binding to VPS29, interactions that facilitate their recruitment to the retromer endosomal retrieval subdomain (Figure 2).

One example is the FAM21 subunit of the WASH complex. This contains an extensive 1100 amino acid unstructured ‘tail’ that contains 21 repeating sequences of an LFa motif (Leu-Phe-acidic residues) [57,58]. Of these, the carboxy-terminal repeat 21 is unique in containing a PL motif (^1330^IFDDPLNAF^1338^) that binds to the hydrophobic groove in VPS29 [54,55]. Two additional LFa motifs bind to two conserved sites in the carboxy-terminal region of VPS35 to provide a three-site mechanism binding to FAM21 and the accompanying WASH complex [54,55]. Notably, although the details are not identical, these interactions in many ways mimic the bipartite interactions of yeast Vps5 with Retromer. In humans, the RAB GAPs TBC1D5, TBC1D1/D4 and TBC1D13, and the RAB GEFs VARP and DENND4A/4C present their own PL motifs for binding to VPS29, which with additional secondary association allows their retromer-driven endosomal recruitment to restrict local RAB7 activation [59–61] and likely RAB10, RAB21, and RAB35 activation [53,56]. Therefore, besides its classical role in sequence-dependent cargo recognition, the human Retromer (and metazoan retromer more broadly) acts as an ‘assembly-point’ to regulate steps at the retrieval subdomain toward the successful biogenesis of a cargo-enriched transport carrier, including the PL motif–independent assembly of the CROP fission factor and EYA proteins [62,63]. The release of VPS29 from the intramolecular PL motif association observed in the yeast pentameric retromer (Figure 1) has empowered the human (and metazoan) Retromer with the ability to act as a focal point for the regulation of localized actin dynamics and RAB switches within the endosomal retrieval subdomain (Figure 2).

Does the VPS29 subunit in Retriever perform the same function as the retromer-associated protein? This turns out not to be the case. Retriever is a stable heterotrimer of VPS26C, VPS35L, and VPS29 [8]. The stability of this complex arises in part from the extended intrinsically disordered amino-terminal region unique to VPS35L (not found in the retromer VPS35 subunit), forming an intramolecular association with the carboxy-terminal region of VPS35L (an extension that is absent in VPS35) [9–11]. This head-to-tail configuration aligns a PL motif within the unstructured amino-terminal region of VPS35L for presentation to the hydrophobic pocket in VPS29 [9–11]. The occlusion of the hydrophobic pocket blocks the association with external PL motifs, so that Retriever is unable to associate with retromer accessory proteins including TBC1D5, VARP, and FAM21 [9]. The structural and functional role of VPS29 is therefore very much retromer-versus-Retriever context specific, and we consider that VPS29 is likely to participate in many more interactions when part of retromer as opposed to Retriever. Indeed, based on relative expression levels, the retromer subunits are typically expressed at a level >10-fold higher than Retriever (see OpenCell database, https://opencell.sf.czbiohub.org). For example, in HEK293T cells VPS35 is at 800 nM and VPS35L at 72 nM, with VPS29A expressed in high-enough excess (approximately 1100 nM) to be present in both complexes without necessarily having to complete between the two. Moreover, this level of VPS29 expression greatly exceeds that of retromer accessory proteins, suggestive of higher-ordered retromer assemblies potentially being heterogeneously decorated with these accessory proteins (expression ranging from VARP at 29 nM to FAM21 and TBC1D13 at around 180 nM). Overall, this serves to further emphasize the uniqueness of retromer beyond its ‘classical’ role in sequence-dependent cargo recognition.

### The ESCPE-1 complex—acquisition of new powers

The ‘separation of powers’ from the confines of the yeast retromer–SNX–BAR pentamer, has allowed human ESCPE-1 to evolve a role in direct sequence-dependent recognition of cargo proteins [47,48]. Here, the PX domains of SNX5 and SNX6 have acquired a 38-amino acid insert that is entirely absent from yeast Vps17, or indeed any other SNX family members [47,48] (Figure 3). This folds into a helix-loop-helix that exposes a hydrophobic groove for association with a bipartite ФxΩxФ(x)nФ sorting motif located in the cytoplasmic facing regions of cargo proteins (x, any amino acid; n, variable linker region) [47,48]. Over 70 human cargos contain this motif and utilize ESCPE-1 for their recycling to the cell surface including CI-MPR, SEMA4C, TRAILR1, and IGF1R [47,48].

Besides acquisition of direct cargo recognition, the other ESCPE-1 subunits, SNX1 and SNX2, have acquired the ability to associate with the cargo adaptor sorting nexin-27 (SNX27) [64–67]. This is mediated through SNX1 and/or SNX2 having acquired acidic-Asp-Leu-Phe (aDLF) motifs in their disordered amino-termini that directly bind into a positive-charged ringed hydrophobic groove in the FERM domain of SNX27 [65–67] (Figure 3). SNX27 contains another modular domain, an aminoterminal PDZ domain, and this conveys an entirely distinct, dual function. First, it contains a binding pocket for cargos that possess a specific subgroup of PDZ-binding motifs at their extreme carboxy-termini—cargos include for example, the β2-adrenergic receptor and the glucose transporter GLUT1 [68,69], and secondly, a solvent-exposed β-hairpin in the SNX27 PDZ domain directly binds to the VPS26A and VPS26B subunits of retromer [70]. Importantly, PDZ-binding motif occupancy of the PDZ domain enhances the affinity for retromer by an order of magnitude [70]. Together, this leads to a model where SNX27 resident on the endosomal membrane captures newly arrived PDZ-binding motif containing cargo and serves to segregate these into the retromer-enriched retrieval subdomain [67]. (A mechanistically distinct but conceptually equivalent event occurs in the transfer of cargo captured by SNX17 into the Retriever-demarcated retrieval subdomaindhere, cargo binding to SNX17 relieves an autoinhibited state thereby promoting association with subdomain-enriched Retriever [71–73].) The direct association of SNX27-retromer to the SNX1/SNX2 subunits of the ESCPE-1 complex serves to transfer captured cargo into forming tubularevesicular transport carriers for onward transport and recycling [65,67]. A further, human-specific (and metazoan-specific) interaction may facilitate these events. The identical hydrophobic groove in the SNX27 FERM domain also binds to aDLF motifs found in the WASH complex subunit FAM21 [55]. Taken alongside the previously discussed direct mechanism of retromer binding to FAM21, these data reveal multiplexed low-affinity interactions within an avidity-based system that incorporates ‘check and balances’ to establish a highly dynamic SNX27-retromer-WASH-ESCPE-1 assembly at the retrieval subdomain. This regulates sequence-dependent cargo recognition and sequestration with localized branched filamentous actin polymerization and the biogenesis of tubular profiles and tubularevesicular transport carriers. None of these associations are observed in yeast, and one could argue that they may not have evolved without the separation of powers from the yeast retromer–SNX–BAR pentamer to the human retromer.

## Conclusions and future directions

The retromer complex is established as a central assembly in eukaryotic cell and developmental biology with a key neuroprotective role in human physiology and age-related [[74,75]] pathophysiology. An everexpanding body of functional data and the identification of an ever-increasing number of retromer accessory proteins is providing ever-greater insights into the biology of this heterotrimeric complex. What is emerging is that Retromer’s associations with accessory proteins in humans and other metazoans is generally mediated through low-affinity, multiplexed interactions that establishes a highly dynamic network of interactions within the retrieval subdomain. Experimentally dissecting this network to obtain a functional insight will therefore be very challenging. What is clear, is that although the SNX-BAR proteins and the retromer complex play key roles in endosomal trafficking, there has been a separation of powers during evolution with the stability of the pentameric yeast retromer–SNX–BAR complex lost in higher eukaryotes. One result of this is that the PL motifebinding pocket in VPS29 is more readily available in higher eukaryotes to associate with key accessory proteins that play essential roles in maintaining RAB GTPase endosomal identity and maturation, and the WASH-mediated F-actin–dependent organization of the endosomal–lysosomal network. When interpreting retromer functions, it is important to consider that besides its classical role in sequence-dependent endosomal cargo retrieval and recycling, the retromer complex has evolved a broader role in organizing and regulating the endosomalelysosomal network. Aside from controlling retrieval and recycling of cargos essential for neuronal synaptic organization and activity and general neuronal health, this broader role of retromer in endosomalelysosomal network organization is likely to be of significance in its neuroprotective role in aged-related Alzheimer’s disease, Parkinson’s disease, and other neurological conditions. Targeting some of these broader roles may provide additional routes for manipulating the neuroprotective role of retromer in age-related neurodegenerative disease.

## Data Availability

No data was used for the research described in the article.
